# BL-Hi-C reveals the 3D genome structure of *Brassica* crops with high sensitivity

**DOI:** 10.1093/hr/uhae017

**Published:** 2024-01-16

**Authors:** Lupeng Zhang, Ranze Zhao, Jianli Liang, Xu Cai, Lei Zhang, Huiling Guo, Zhicheng Zhang, Jian Wu, Xiaowu Wang

**Affiliations:** State Key Laboratory of Vegetable Biobreeding, Institute of Vegetables and Flowers, Chinese Academy of Agricultural Sciences, Beijing 100081, China; State Key Laboratory of Vegetable Biobreeding, Institute of Vegetables and Flowers, Chinese Academy of Agricultural Sciences, Beijing 100081, China; State Key Laboratory of Vegetable Biobreeding, Institute of Vegetables and Flowers, Chinese Academy of Agricultural Sciences, Beijing 100081, China; State Key Laboratory of Vegetable Biobreeding, Institute of Vegetables and Flowers, Chinese Academy of Agricultural Sciences, Beijing 100081, China; State Key Laboratory of Vegetable Biobreeding, Institute of Vegetables and Flowers, Chinese Academy of Agricultural Sciences, Beijing 100081, China; State Key Laboratory of Vegetable Biobreeding, Institute of Vegetables and Flowers, Chinese Academy of Agricultural Sciences, Beijing 100081, China; State Key Laboratory of Vegetable Biobreeding, Institute of Vegetables and Flowers, Chinese Academy of Agricultural Sciences, Beijing 100081, China; State Key Laboratory of Vegetable Biobreeding, Institute of Vegetables and Flowers, Chinese Academy of Agricultural Sciences, Beijing 100081, China; State Key Laboratory of Vegetable Biobreeding, Institute of Vegetables and Flowers, Chinese Academy of Agricultural Sciences, Beijing 100081, China

## Abstract

High-throughput Chromatin Conformation Capture (Hi-C) technologies can be used to investigate the three-dimensional genomic structure of plants. However, the practical utility of these technologies is impeded by significant background noise, hindering their capability in detecting fine 3D genomic structures. In this study, we optimized the Bridge Linker Hi-C technology (BL-Hi-C) to comprehensively investigate the 3D chromatin landscape of *Brassica rapa* and *Brassica oleracea*. The Bouquet configuration of both *B. rapa* and *B. oleracea* was elucidated through the construction of a 3D genome simulation. The optimized BL-Hi-C exhibited lower background noise compared to conventional Hi-C methods. Taking this advantage, we used BL-Hi-C to identify *FLC* gene loops in *Arabidopsis*, *B. rapa*, and *B. oleracea*. We observed that gene loops of *FLC2* exhibited conservation across *Arabidopsis*, *B. rapa*, and *B. oleracea*. While gene loops of syntenic *FLCs* exhibited conservation across *B. rapa* and *B. oleracea*, variations in gene loops were evident among multiple paralogs *FLCs* within the same species. Collectively, our findings highlight the high sensitivity of optimized BL-Hi-C as a powerful tool for investigating the fine 3D genomic organization.

## Introduction

The organization of DNA into chromatin in the nucleus of eukaryotic cells affects transcription, DNA replication, and other nuclear functions [1]. High-throughput Chromosome Conformation Capture (Hi-C) technology enables the simultaneous interrogation of all contact loci, yielding a comprehensive genome-wide interaction map through high-throughput sequencing [2]. The application of Hi-C in plant research provides valuable insights into 3D genome organization. Chromosomes occupy distinct nuclear territories, referred to as chromosome territories (CTs) [3], each exhibiting unique morphologies including Rabl, Rosette, and Bouquet configurations. The construction of a 3D genome model has revealed that the 3D genome feature of rice assumes a non-Rabl configuration [4]. In *Arabidopsis*, the centromeric region is concerned, while the euchromatin emanates outward representing the Rosette configuration [5]. Many plants exhibit compartments and domains similar to topologically associated domains (TADs) [6–9]. *Brassica rapa* and *Brassica oleracea* were found to have conserved compartments and TADs [8]. Recent studies showed that 3D chromatin architecture contributed to heterosis in *Brassica napus* [10]. In *Brassica*, genes with higher numbers of conserved noncoding sequences (CNSs) are more likely to contact distant genes [11].

Although Hi-C technology harbors significant potential for uncovering the landscape of 3D genomes, its widespread application is impeded by high background noise. Notably, Hi-C has not been extensively employed to investigate gene loops, a crucial structure for orchestrating gene expression [12]. The proximity ligation strategy employed in Hi-C can introduce noise during the process [13]. Extracting reliable interactions from the high-noise Hi-C data often needs deep sequencing and substantial bioinformatics efforts [14]. In efforts to reduce background noise, several advanced Hi-C methods, including HiCAR [15], ChIATAC [16], Hi-TrAC [17], and Hi-Tag [18] have been developed. However, these methods primarily capture interactions associated with histone modification or accessible chromatin. Bridge Linker-Hi-C (BL-Hi-C) technology emerged as a powerful tool for exploring the chromatin architecture of the whole genome [19]. BL-Hi-C has been used in identifying key transcription factors such as myogenic differentiation 1 (MyoD) [20] and CCCTC-binding factor (CTCF) [21]. However, the original research did not discuss whether BL-Hi-C has the potential to detect gene loops [22].

*Brassica* species, major vegetables worldwide, are closely related to *Arabidopsis* [23]. It has been reported that expression of genes in *Arabidopsis* is regulated by gene loops. *FLOWERING LOCUS C* (*FLC*) acts as a central repressor of flowering [24]. Previous 3C experiments demonstrated the *FLC* promoter contacts with downstream regions [25], with Hi-C yielding a similar but less evident result [26]. *FRIGIDA* (*FRI*) binding on *FLC* increases physical contact between promoter and downstream, which promotes the expression of *FLC* [27]. The genomes of *Brassica* species underwent a lineage-specific whole-genome triplication (WGT) event followed by diploidization [28]. In both *B. rapa* and *B. oleracea*, there are four *FLC* homologs: *FLC1*, *FLC2*, *FLC3*, and *FLC5* [29]. Previous studies have shown that the function of *FLC*s is similar to that of *Arabidopsis* [13]. However, it remains unknown whether *FLC* gene loops are conserved in *Brassica*.

In this study, we demonstrated the excellent performance of BL-Hi-C in the genome-wide profiling of chromatin interactions in *B. rapa* and *B. oleracea*. Through the construction of a 3D structure model, we illustrated the Bouquet configuration of both *B. rapa* and *B. oleracea*. In comparison with traditional Hi-C, BL-Hi-C displayed reduced background noise, thereby enhancing its reliability in detecting gene loops. We observed high conservation in gene loops between *FLC2s* of both *B. rapa* and *B. oleracea* and those of *Arabidopsis*. While gene loops in syntenic *FLC*s of *B. rapa* and *B. oleracea* were conserved, variations in gene loops were evident among paralogous *FLC* copies within the same species. Our findings highlight the potential of BL-Hi-C as a valuable tool for investigating small 3D genome organization in plants.

## Results

### Modification of BL-Hi-C for plant species

**Figure 1:**
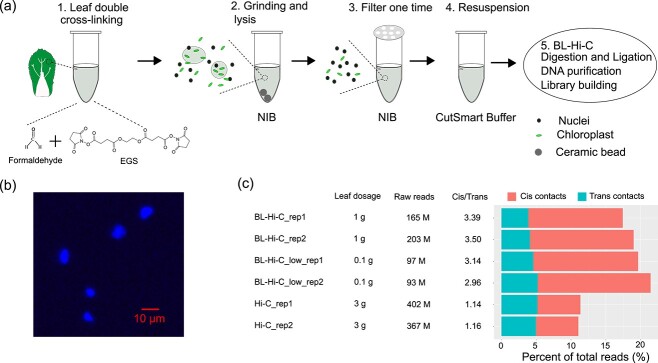
Schematic of BL-Hi-C method. (**a**) The nuclei extraction procedure includes double cross-linking, grinding and lysis, filter, and resuspension. (**b**) The nuclei isolated were stained with 4′,6-diamidino-2-phenylindole (DAPI) and observed under a fluorescence microscope. (**c**) Efficiency comparison of BL-Hi-C, low input BL-Hi-C and Hi-C in *Brassica rapa*. The *cis* contacts refer to intrachromosomal interactions, and the *trans* contacts refer to interchromosomal interactions.

Original BL-Hi-C requires a minimum of 0.5 million cells for the identification of chromosome interactions. However, counting the number of nuclei under the microscope can be challenging due to the presence of impurities in the nuclei isolated from plant tissue. Instead, we used a certain amount of leaf tissue to estimate the number of nuclei that would satisfy the requirements for library construction. Our results showed that 1 g fresh leaves of *B. rapa* and *B. oleracea* was sufficient to obtain enough nuclei. Traditional Hi-C usually requires 3 ~ 5 g of fresh leaf. Reduction in starting material not only simplifies the experimental procedure but also allows the experiment to be completed within 2.5 days in a 1.5 ml tube (Fig. 1a). Additionally, by reducing the reaction volume, the cost of library generation was able to decrease to as low as $92 per sample, which is approximately one-third the cost of traditional Hi-C (Table S2, see online supplementary material).

We simplified the nuclei extraction procedure. In BL-Hi-C, the digestion and ligation processes are completed within the intact nuclei. Previous nuclei isolation protocols require a significant amount of tissue and long duration for completion because of grinding samples manually under liquid nitrogen and the multiple purification steps. We simplified the nuclei extraction procedure by grinding the leaf tissue with an electric grinder (step 2, Fig. 1a) and reducing the filtration step to only once (step 3, Fig. 1a). Observed under a fluorescence microscope, it showed that most of the nuclei were intact enough to meet BL-Hi-C requirements (Fig. 1b). After resuspension in CutSmart Buffer (step 4, Fig. 1a), nuclei were used to construct the library following the standard BL-Hi-C procedure (step 5, Fig. 1a).

To ensure that all steps of the BL-Hi-C library construction had been performed correctly, DNA fragments from each step were purified and analysed by gel electrophoresis (Fig. S1a, see online supplementary material). A typical smear of DNA fragments was observed after chromatin digestion using the restriction enzyme *Hae* III. After ligating digested chromatin to linkers, the DNA fragments were aggregated. These results indicated that the genome digestion and the proximity ligation were completed.

### 3D genome organization captured by BL-Hi-C

Utilizing the identical analysis pipeline, we conducted a comparative assessment of the 3D genomic data of *B. rapa* generated through Hi-C ^8^ and BL-Hi-C. The ratio of valid contacts concerning sequencing reads was notably higher in BL-Hi-C (18%) compared to Hi-C (11%) (Fig. 1c). After removing fragments with interaction distances less than 1 Kb, BL-Hi-C exhibited a cis:trans ratio of 3.45 higher than 1.2 in Hi-C (Fig. 1c). BL-Hi-C demonstrated the identification of chromatin contacts across a broad distance range with an efficiency comparable to that of the Hi-C method (Fig. S1b, see online supplementary material). To evaluate the robustness of BL-Hi-C, we conducted the assay using leaves of *B. oleracea*, resulting in a total of 28 million valid contacts, occupying 29% of total reads (Table S1, see online supplementary material). The cis:trans ratio in BL-Hi-C for *B. oleracea* was 5.1, surpassing that in Hi-C (1.13) [8]. These outcomes from the *B. oleracea* experiment once again emphasized the excellent performance of BL-Hi-C.

**Figure 2:**
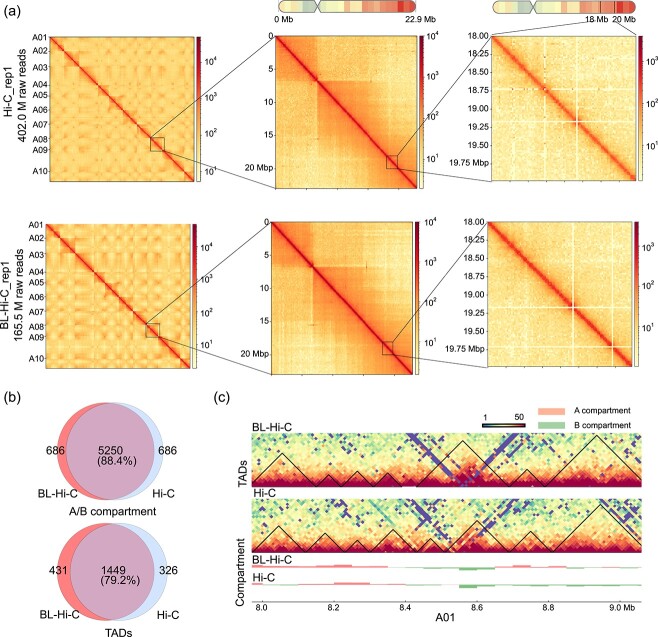
Results comparison between BL-Hi-C and Hi-C in *Brassica rapa*. (**a**) Heatmaps reconstructed using data generated from Hi-C and BL-Hi-C. The resolution was set as 500 Kb for the entire genome, 150 Kb for the A08 chromosome, and 20 Kb for the local regions. (**b**) Overlap of compartments and TADs between BL-Hi-C and Hi-C. (**c**) Part of genome browser images showing compartments and TADs structure detected on A01 by BL-Hi-C and Hi-C.

To validate the ability of BL-Hi-C in detecting crucial aspects of genome architecture, we compared the contact heat maps generated by the two methods (Fig. 2a). Despite being derived from distinct sequencing depths in *B. rapa*, the heat maps exhibited similar interaction patterns at multiple scales, including the genome, chromosome, and local levels. In *B. oleracea*, BL-Hi-C and Hi-C also showed similar interaction signals across the whole genome (Fig. S2a and b, see online supplementary material).

In 3D genomics, compartments and TADs play critical roles in regulating gene expression. We compared the compartments and TADs obtained from BL-Hi-C and Hi-C employing the same analysis pipeline. The BL-Hi-C and Hi-C displayed a high degree of consistency regarding their A/B compartments (Fig. 2b). The TADs overlapped more than 80% between BL-Hi-C and Hi-C were considered overlapped TADs. Among the 1880 TADs identified from 368 million raw reads in the BL-Hi-C, 1449 (77.1%) were shared in Hi-C. The consistence of TAD structure between BL-Hi-C and Hi-C was clearly shown in the visualization (Fig. 2c). Taken together, BL-Hi-C faithfully captures TADs and compartments in the plants.

**Figure 3:**
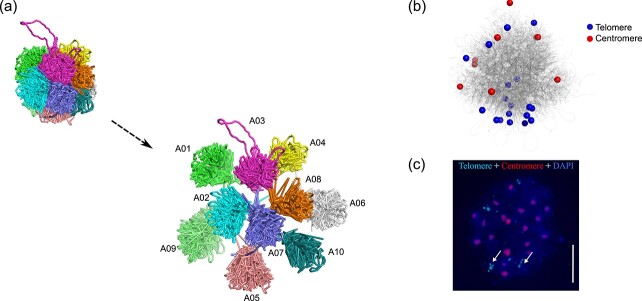
Reconstructed particle-on-a-string 3D genomes of *Brassica rapa*. (**a**) 3D organization with expanded views of the separate chromosome territories. (**b**) Spatial distribution of telomeres and centromeres of ten chromosomes in the 3D genome. The blue sphere indicates the telomeres, and the red sphere indicates centromeres. Each particle equals ten kilobase pairs. (**c**) Chromatin was stained blue with DAPI. Fluorescence in situ hybridization was performed with probes specific for centromeres (red) and telomeres (blue). Arrows indicate clusters of multiple telomeres. Scale bar, 2 μm.

To investigate the chromosome organization in *B. rapa* nuclei, we constructed 3D genome structures (Fig. 3a) using BL-Hi-C. Our analysis of the 3D genome architecture of *B. rapa* nuclei illustrated that each chromosome occupied an exclusive region within the nuclei, supporting the concept of ‘chromosome territory’. Intrigued by the morphology of *B. rapa* nuclei, we labeled the 10 centromeres and 20 telomeres on the 3D structural model of the genome with different colors. The telomeres were found to be localized close to each other, while the centromeres were located at the periphery of the nuclei (Fig. 3b). We observed similar results in *B. oleracea* as well (Fig. S3, see online supplementary material). Fluorescence *in situ* hybridization also supported clusters of telomeres (Fig. 3c). These results demonstrate the presence of the Bouquet configuration in *B. rapa* and *B. oleracea* leaf nuclei.

### Low background noise of BL-Hi-C

**Figure 4:**
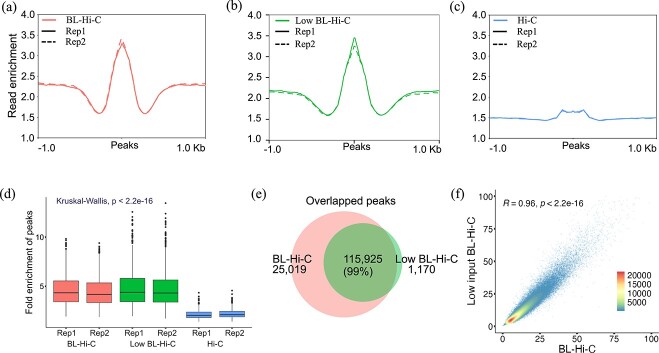
Comparison of BL-Hi-C, low input BL-Hi-C and Hi-C signal-to-noise ratio. (**a**–**c**) Reads enrichment around peaks. (**d**) Boxplots showing the fold enrichment of peaks (*n* = 1000). (**e**) Venn diagram showing the overlapped peaks of BL-Hi-C and low input BL-Hi-C. (**f**) Scatter plots of the peak intensity between BL-Hi-C and low input BL-Hi-C (*n* = 144 832). The R-value is the Spearman’s correlation coefficient.

To assess the signal-to-noise of BL-Hi-C relative to Hi-C, we compared peaks generated by BL-Hi-C and Hi-C in *B. rapa*. The read enrichment of peaks in BL-Hi-C was 3.5, surpassing the value of 1.7 observed in Hi-C (Fig. 4a and c). To remove the effect of background we calculated the fold enrichment of peaks (read coverage in peaks/average read coverage). The average fold enrichment was 4.7 for BL-Hi-C peaks, which was significantly higher than 2.1 identified from Hi-C peaks (*P* < 2.2e-16, Fig. 4d). Similar interaction signals were also observed in *B. oleracea* (Fig. S2c, see online supplementary material). These results demonstrated that BL-Hi-C had the advantage over Hi-C in signal-to-noise ratio for chromosome conformation capture.

To test the feasibility of a small amount of sample using BL-Hi-C, we built the BL-Hi-C library using only 100 mg of leaf. We found 99% of the low-input BL-Hi-C peaks overlapped with the BL-Hi-C peaks (Fig. 4e). A high correlation (Spearman’s correlation = 0.96) was also observed between low-input BL-Hi-C and BL-Hi-C in peak intensity (Fig. 4f). Low input BL-Hi-C read enrichment and fold enrichment of peaks were 3.5 and 4.7, consistent to BL-Hi-C (Fig. 4b and d). These results confirmed that decreasing the input amount to as low as 100 mg tissue did not reduce the robustness of BL-Hi-C.

### Gene loops detected by BL-Hi-C

Conserved gene loops between *FLCs* of *Brassica* and *Arabidopsis* were detected from our BL-Hi-C analysis. Previous studies have reported the presence of a gene loop between the *FLC* promoter and the downstream in *Arabidopsis* [25, 26]. Our BL-Hi-C analysis for *Arabidopsis* revealed distinct loops, including promoter-intron, intron-downstream, and intron-intron loops, in addition to the previously reported promoter-downstream loops in *FLC* (Fig. 5a). For *B. rapa* and *B. oleracea*, our BL-Hi-C analysis unveiled gene loops in *FLC1*, *FLC2*, and *FLC3*, encompassing loop types of promoter-downstream, promoter-intron, and intron-downstream (Fig. 5b–e). However, such gene loops were not observed in the Hi-C analysis (Fig. S4, see online supplementary material). Gene loop anchors were identified in the 3′ and 5′ UTR regions of both *BrFLC2* and *BoFLC2*, located in the syntenic region with *Arabidopsis FLC* (Fig. 5c). Both *BrFLC2* and *BoFLC2* lack a gene loop anchor in their first introns due to the anchor sequence being lost compared to *AtFLC* (Fig. 5c). These results indicate that *Arabidopsis FLC* and *Brassica FLC2s* are conserved not only in sequence but also in 3D gene structure. Nevertheless, gene loops detected at *FLC1*, *FLC3*, and *FLC5* differed from *Arabidopsis FLC* (Fig. 5b, d and e).

**Figure 5:**
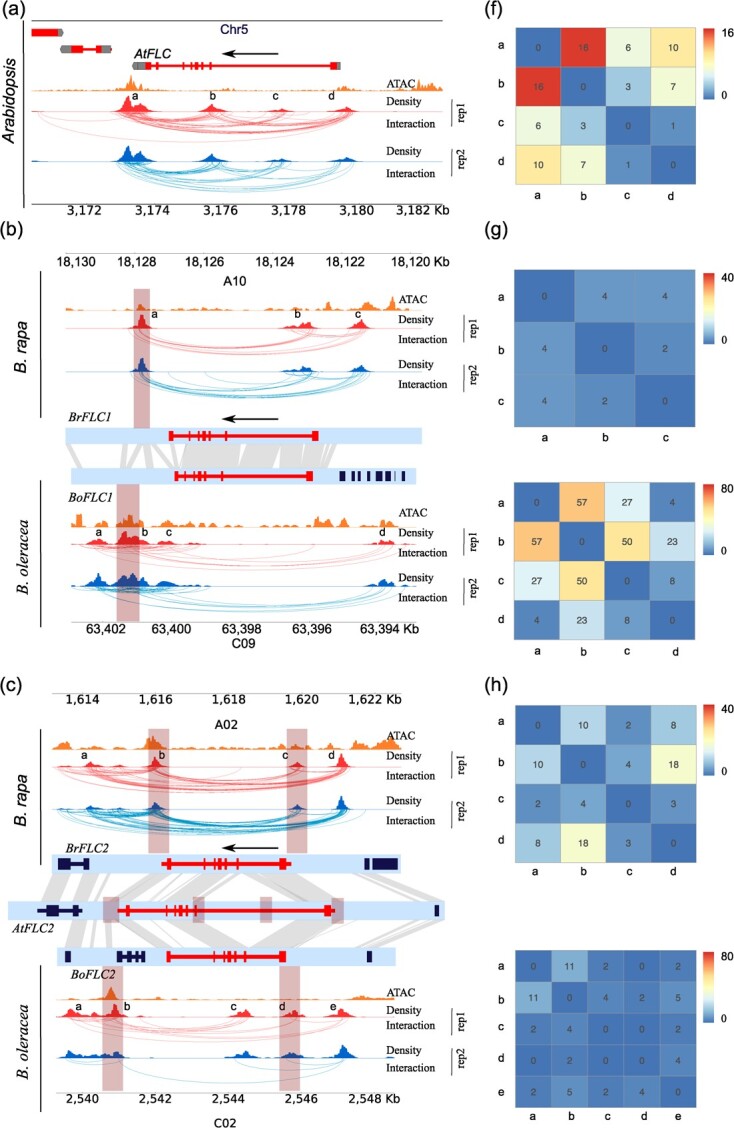
Continues

**Figure 5:**
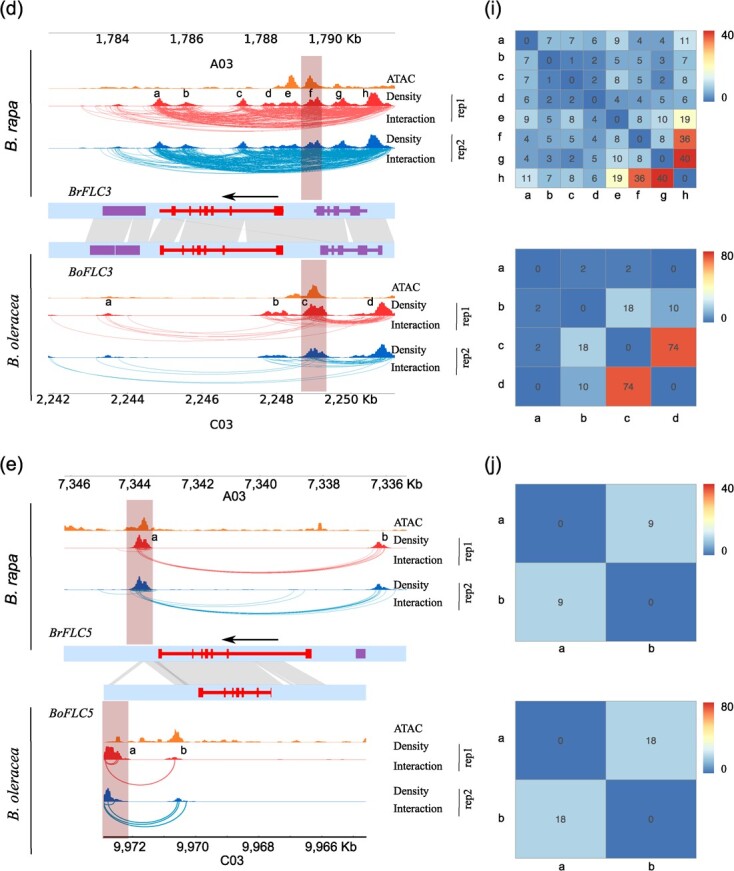
Comparison of *FLC* gene loops between *Brassica rapa* and *Brassica oleracea*. (**a**) Gene loops at *Arabidopsis AtFLC* (*AT5G10140*). (**b**–**e**) Gene loops at *Brassica FLCs* and syntenic analysis using ±3 Kb surrounding regions of *FLC*s. Gray lines connect syntenic regions between *B. rapa* and *B. oleracea*. The brown box indicates the anchor region of the gene loop. There are four *FLC* homologs in *B. rapa* including *BrFLC1* (*BraA10g027790.3.5C*), *BrFLC2* (*BraA02g003370.3.5C*), *BrFLC3* (*BraA03g004250.3.5C*), *BrFLC5* (*BraA03g015930.3.5C*), and four *FLC* homologs in *B. oleracea* including *BoFLC1* (*BolC09g062620.2 J*), *BoFLC2* (*BolC02g004040.2 J*), *BoFLC3* (*BolC03g004550.2 J*), *BoFLC5* (*BolC03g017850.2 J*). (**f**–**j**) Gene loop strength of *Arabidopsis*, *B. rapa,* and *B. oleracea* was shown in heatmap.

*Brassica* underwent a genome triplication event after its divergence from *Arabidopsis*. Genes associated with Gene Ontology terms related to responses to environmental factors, including salt, cold, osmotic stress, light, etc., were over-retained in *Brassica* genomes [28]. *Brassica FLCs* function similarly to *Arabidopsis* in the regulation of flowering [14]. We utilized *FLCs* as an example to analyse the conservation of gene loops among paralogs in *Brassica* species. Our analysis uncovered loop anchors downstream of both *BrFLC1* and *BoFLC1*. However, *BrFLC1* exhibited a promoter-downstream loop and an intron-downstream loop, whereas *BoFLC1* only had a promoter-downstream loop (Fig. 5b). For *BrFLC2* and *BoFLC2*, in addition to identical loop anchor positions with *Arabidopsis*, *BoFLC2* introns contained loop anchors, but this was not observed in *BrFLC2* (Fig. 5c). The *BrFLC3* gene body exhibited numerous interactions forming a domain-like structure (Fig. 5d). Loop anchors were identified downstream of *BrFLC5* and *BoFLC5*, forming a promoter-downstream loop only in *BrFLC5*, not in *BoFLC5* (Fig. 5e). Comparing gene loops of different copies of *FLC* from the same species revealed significant variations in anchor locations. Notably, these loop anchors exhibited substantial overlap with the peaks detected in ATAC-seq, illustrating the association of gene loops with transcription factor occupancy (Fig. 5b–e). Despite some differences in gene loops between *B. rapa* and *B. oleracea*, the analysis of syntenic genes underscores similarity in their gene loops.

To further quantitatively analyse gene loops, we define a loop strength as the observed interactions in gene loops divided by that ±100 Kb surrounding regions. For *AtFLC*, the strength of the promoter-downstream loop (a, d) was 10, and a promoter-intron loop (a, b) reached up to 16, the highest among the loops detected for *AtFLC*. The intron-intron loop (b, c) and intron-downstream loop (c, d) were only 3 and 1 in strength (Fig. 5f). For *BrFLC1*, strengths of the promoter-downstream loop (a, c) and intron-downstream loop (a, b) were both 4, lower than the 23 of *BoFLC1* promoter-downstream loop (b, d) (Fig. 5g). Strengths of the promoter-downstream loop for *BrFLC2* (b, c) and *BoFLC2* (b, d) were 4 and 2, representing conserved gene loops (Fig. 5h). In *BrFLC2*, the strongest gene loop was another promoter-downstream loop (b, d), with a strength of 18. The strength of the promoter-downstream loop was 9 for *BrFLC3* (a, e) (Fig. 5i). We noticed that strong gene loops present in the promoters of *BrFLC3* (f, h) and *BoFLC3* (c, d), with strength of 36 and 74, indicating gene loops in promoter regions might regulate *FLC3* expression. The diverse gene loops might indicate their fine-tuning role in the regulation of *FLC* gene expression.

## Discussion

Over the past decade, traditional Hi-C has significantly advanced research on plant 3D genomes [22]. In the present study, we simplified the nuclei extraction step to enable efficiently utilizing BL-Hi-C in plant research. We have successfully applied nuclei extraction method to *Arabidopsis, B. rapa* and *B. oleracea* demonstrating robustness of the method. BL-Hi-C can be performed with leaf sample amount as low as 100 mg, which is only 1/30 of Hi-C. Importantly, we found BL-Hi-C is high signal-to-noise and can detect gene loops.

Low-input BL-Hi-C enables the analysis of samples with limited starting material, such as pollen and shoot tips, which have received relatively little attention in research. With the rapid advancement of sequencing technology, large-scale resequencing and transcriptome sequencing have become prevalent in plant research [29–31]. As epigenetic regulation emerges as a crucial factor in development and environmental adaptation, the exploration of 3D genomics is considered essential for uncovering additional insights beyond resequencing and transcriptome analyses [32, 33]. Genome-Wide Association Studies (GWAS) and population transcriptome analyses have successfully identified numerous regulatory elements [34, 35]. Hi-C revealed connection between regulatory elements and target genes [36]. However, due to the limited number of samples, it has been challenging to fully elucidate the relationship between regulatory elements and target genes [37]. Owing to complex library construction procedures and high costs, the current number of samples analysed in 3D genomes is limited to less than 30 [37, 38]. By reducing start material, our method makes experimental processes easy to conduct and the cost is much cheaper (Table S2, see online supplementary material). This provides an option for conducting 3D-genomics analysis in a population scale along with resequencing and transcriptome analysis.

Previous investigations into the 3D genomes of plants have predominantly focused on compartments and topologically associating domains (TADs), with less attention given to chromatin loops. In mammalian organisms, chromatin loops are believed to play a pivotal role in facilitating specific interactions and communication between enhancers and promoters [39]. However, the identification of chromatin loops in plants using Hi-C technology poses challenges due to high background noise, unless ultra-deep Hi-C datasets containing billions of contact reads are employed [40]. The application of ChIA-PET technology, characterized by reduced background noise, proves to be efficient for investigating chromatin loops even at lower sequencing depths [41]. Nevertheless, ChIA-PET analysis is limited to specific protein-mediated chromatin loops. Our results indicate that BL-Hi-C has an advantage over ChIA-PET in terms of independence of antibody enrichment. In the future, BL-Hi-C could be employed to identify chromatin loops as an alternative method to ChIA-PET.

Gene loops have emerged as dynamic mechanisms regulating the expression of specific genes [12]. It was observed that two regions flanking the *FLC* gene body form a loop that promotes expression [25]. While gene loops are primarily studied using 3C, which is only feasible for investigating a limited number of loops. BL-Hi-C, due to its low background noise, can successfully detect gene loops genome-wide at low sequencing depths. In previous studies, 162 million valid contacts were used to identify the *FLC* promoter and downstream gene loops using traditional Hi-C [26]. Our BL-Hi-C analysis identified *Arabidopsis FLC* gene loops between the promoter and downstream using only 27 million valid contacts (Table S1, see online supplementary material). According to a recent quantitative 3C analysis, the strongest interactions were found between promoters and downstream in *FLC* gene [27]. However, our BL-Hi-C data revealed the strongest interactions between the promoter and the first intron. This difference may arise from variations in plant conditions, given that gene loops are dynamically altered. *Brassica* species possess four copies of *FLC*, three originating from the whole genome triplication (WGT) event, and one from α-duplication [42]. Among the four copies, we found that gene loops of *FLC2* in both *B. rapa* and *B. oleracea* were similar to those of *Arabidopsis*. Additionally, we observed that gene loops of *FLC* copies in a syntenic relationship between *B. rapa* and *B. oleracea* exhibit higher similarity in terms of anchor location and gene loop types than those between paralogues. Considering that gene loops are a regulatory mechanism for gene expression, this result suggests that syntenic genes share more similar fine-tuning mechanisms, while paralogues diverge not only in their sequence but also in the 3D local organization of chromatin. It was found that highly expressed genes tend to form gene loops [26]. Among the four copies of *B. rapa*, we discovered that *BrFLC3* had the most gene loops, while *BrFLC5* had the least. Long-read RNA sequencing revealed that the expression of *BrFLC3* was the highest and *BrFLC5* was lowest than that of other copies in the accession Chiifu [43]. These results imply that gene loops might play important roles in *FLC* expression regulation.

In summary, we showed BL-Hi-C technology exhibits lower background noise compared to that of Hi-C, which enable BL-Hi-C detect gene loops. We found gene loops of *FLC2* displaying conservation across *Arabidopsis*, *B. rapa*, and *B. oleracea*. Although gene loops of syntenic *FLCs* were conserved across *B. rapa* and *B. oleracea*, gene loops varied among paralogous *FLCs* within a single species. These results indicate BL-Hi-C is a powerful method for analysing fine 3D genomes.

## Materials and methods

### Plant material

*Arabidopsis* Col-0 seeds were surface-sterilized by treatment with sodium hypochlorite, washed, and then sown in sterile 1/2 MS medium. After 2 weeks the seedlings were transferred to soil in a greenhouse at 23°C with a 16 h photoperiod. After 2 weeks, the harvestable young leaves were used for BL-Hi-C. *B. rapa* (ssp. pekinensis, acc. Chiifu) and *B. oleracea* (acc. JZS) seeds were cultivated in Petri dishes at a room temperature (25°C) for 14 h, and the seedlings were transferred to soil in a greenhouse at 23°C with a 16 h photoperiod. After 1 month, the harvestable young leaves were used for BL-Hi-C.

### Experimental protocol for plant BL-Hi-C

#### Double cross-linking

For *B. rapa*, 2 g of material of each sample was collected in the 50 ml tube, adding 20 mL of NIB (20 mM Hepes (pH 8), 250 mM sucrose, 1 mM MgCl_2_, 0.5 mM KCl, 40% glycerol, 0.25% Triton X-100, 0.1 mM phenylmethanesulfonylfluoride (PMSF), 0.1% 2-mercaptoethanol), 20 ml of 4% formaldehyde (Sigma, #F8775), and 100 μl 0.15 M of EGS (Thermo, #21565) to submerged leaves. Then, the tube was placed in a desiccator, applying vacuum for 1 h. After cross-linking, the remaining formaldehyde was sequestered by adding 2680 μl of 2 M glycine (Sigma, G7126) and continued applying the vacuum for 5 minutes. Next, the NIB/formaldehyde mixture was removed, washing the leaf using ddH2O.

#### Nuclei extraction

Samples were ground in liquid nitrogen to fine powder and then lysed in NIB. The mixture was aliquotted into two tubes and filtered once with Miracloth (Millipore, #475855). After spinning down the filtrate at 3000 g at 4°C for 15 mins, the supernatant was removed. Subsequently, the nuclei were resuspended in 1.3 × CutSmart Buffer and spun down at 1900 *g* at 4°C for 5 min.

#### Nuclei lysis and restriction digestion

Nuclei were then resuspended in 50 μl of 0.5% SDS and incubated at 62°C for 10 mins on the thermomixer (Thermo USA), shaking at 900 r.p.m. After nuclei lysis, 145 μl of ddH_2_O and 25 μl of 10% (v/v) Triton X-100 were added into the tube to quench the SDS reaction. The mixture was then gently shaken for 15 minutes at 37°C. Then, 25 μl of 10 × CutSmart Buffer and 10 μl of *Hae III* (NEB, #R0108L) were added to each tube, and the tubes were incubated at 37°C for 12 h with rotation at 900 r.p.m. Finally, 2.5 μl of 100 mM dATP solution and 2.5 μl of Klenow Fragment (3′- > 5′ exo-) (NEB, #M0212L) were added to the mixture, and the mixture was incubated for 40 minutes at 37°C with rotation at 900 r.p.m.

#### Proximity ligation

After restriction enzyme digestion, the linker (F:pCGCGATATC/iBiodT/TATCTGACT, R:pGTCAGATAAGATATCGCGT) was ligated to the digested chromatin. In each tube, 750 μl of ddH_2_O, 120 μl of 10× T4 DNA ligase buffer, 100 μl of 10% (v/v) Triton X-100, 5 μl of T4 DNA ligase (NEB, #M0202L), and 4 μl of 200 ng/μl linker were added to the 260 μ l of digested chromatin and mixed thoroughly. The mixture was then incubated at 16°C for 4 h with rotation at 900 r.p.m. After linker ligation, chromatin DNA-protein complexes were centrifuged at 3500 g for 5 minutes at 4°C, and the supernatant was discarded. Next, the pellets were resuspended in 309 μl of ddH_2_O, 35 μl of Lambda Exonuclease Buffer, 3 μl of Lambda Exonuclease (NEB, #M0262L), and 3 μl of Exonuclease I (NEB, #M0293L). The mixture was then incubated at 37°C for 1 hour at 900 r.p.m.

#### DNA purification

After proximal ligation, 45 μl of 10% SDS and 55 μl of 10 mg/ml proteinase K (Solarbio, #P1120) was added to the tube, incubated the nuclei at 60°C for about 3 hours to reverse crosslinking. After incubation, 450 μl of phenol:chloroform:isoamyl alcohol (25:24:1) was added to the tube, which was shaken vigorously and then centrifuged for 15 min at 14000 r.p.m. Next, 400 μl of supernatant was transferred into a new tube. DNA was precipitated with 400 μl of isopropanol, 40 μl of 3 M sodium acetate (pH 5.2), and 4 μl of Dr. GenTLE Precipitation Carrier (Takara, #9094) and centrifuged for 15 min at 12000 r.p.m. The precipitated DNA was washed once with 80% ethanol and dissolved in 40 μl of 0.1 × TE Buffer.

#### Library generation

The Bioruptor (Diagenode) was used to break the DNA into 300–500 bp using the following settings: Duty cycle 32; 30 s on, 30 s off. And then 1.2 × Ampure XP beads (Beckman, #A63881) were used to purify the DNA fragments. After the pull-down of biotin-labeled DNA reads (Thermo, #11205D), the DNA Library Prep kit (Enzyme, ND608) for Illumina was used to complete DNA damage repair, end-repair, adaptor ligation, and PCR library amplification. After 12 amplification cycles, the DNA product was purified by 1 × Ampure XP beads for deep sequencing.

### Experimental protocol for low-input plant BL-Hi-C

#### Double cross-linking

For *Arabidopsis*, *B. rapa*, and *B. oleracea*, 0.1 g of material of each sample was collected in the 1.5 ml tube, adding 500 μl of nuclei isolation buffer (NIB: 20 mM Hepes (pH 8), 250 mM sucrose, 1 mM MgCl_2_, 0.5 mM KCl, 40% glycerol, 0.25% Triton X-100, 0.1 mM phenylmethanesulfonylfluoride (PMSF), 0.1% 2-mercaptoethanol), 500 μl of 4% formaldehyde (Sigma, #F8775), and 10 μl 0.15 M of EGS (Thermo. #21565) to submerged leaves. Then, the tube was placed in a desiccator, applying vacuum for 1 h. After cross-linking, the remaining formaldehyde was sequestered by adding 67 μl of 2 M glycine (Sigma, G7126) and continued applying the vacuum for 5 minutes. Next, the NIB/formaldehyde mixture was removed, washing the leaf using ddH2O.

#### Nuclei extraction

The electric grinder was used to grind the leaf with the parameter 60 s, 60 Hz, 4 cycles, and then lysed in NIB. The mixture was transferred into new tubes and filtered once with Miracloth (Millipore, #475855). After spinning down the filtrate at 3000 *g* at 4°C for 15 mins, the supernatant was removed. Subsequently, the nuclei were resuspended in 1.3 × CutSmart Buffer and spun down at 1900 *g* at 4°C for 5 min. The next procedure was the same as that described above for the plant BL-Hi-C protocol.

### Analysis of BL-Hi-C

The trimLinker of ChIA-PET2 (v0.9.3) [44] was used to filter the linker, and HiC-Pro (v3.0.0) [45] was used to align the sequence to the reference genome (*Arabidopsis* TAIR 10; *B. rapa* v3.0; http://39.100.233.196:82/download_genome/Brassica_Genome_data/Brara_Chiifu_V3.0/Brapa_sequence_v3.0.fasta.gz; *B. oleraceaa* v2.0; http://39.100.233.196:82/download_genome/Brassica_Genome_data/Braol_JZS_V2.0/Brassica_oleraceaa_JZS_v2.fasta.gz). Reads with low mapping quality (MAPQ <10) were filtered out and reads with the same coordinate on the genome or mapped to the same digestion fragment were removed. The ICE method was applied to normalize the interaction matrix for different resolutions (10 Kb, 20 Kb, 40 Kb, 150 Kb, and 500 Kb). HiCExplorer (v2.1.4) [46] was used to convert normalized matrix data into h5 format and other formats for further analysis. We combined biological replicate data for analysis of compartments and TADs. A/B compartments were determined by Juicer (v1.9.9) [47] at 50 Kb resolution. The TAD boundaries were analysed by HiTAD at 10 Kb resolution (v0.4.2) [48]. According to the previous research in rice single-cell [4], 10 kilobase pixels were chosen as parameters for Nuc_dynamic (https://github.com/tjs23/nuc_dynamics) analysis. A total of 500 000 valid contacts were randomly selected to construct the 3D model three times. Simulated annealing was calculated by the Nuc_dynamic software (parameter: -s 8 2 1 0.5 0.2 0.1 0.05 0.02 0.01) to create a PDB file for viewing the 3D genome structures in pymol (v4.60). Peaks were called from valid contacts by MACS2 (v2.2.7.1) with default parameters.

### Gene loop analysis

*B. rapa* and *B. oleracea FLC* syntenic analysis using MicroSynteny model of the BRAD website [49]. We define a loop strength as the observed interactions in gene loops divided by the background interactions. The interactions in ±100 Kb surrounding regions were defined as background.

S = Obs/Background*10000.

## Supplementary Material

Web_Material_uhae017
