# Supplementary material for: BL-Hi-C reveals the 3D genome structure of Brassica crops with high sensitivity
Source: Hortic Res. 2024 Jan 16;11(3):uhae017. doi: 10.1093/hr/uhae017 (PMC10923644; doi:10.1093/hr/uhae017)
Supplement: Web_Material_uhae017 [file web_material_uhae017.zip › supplementary.docx]

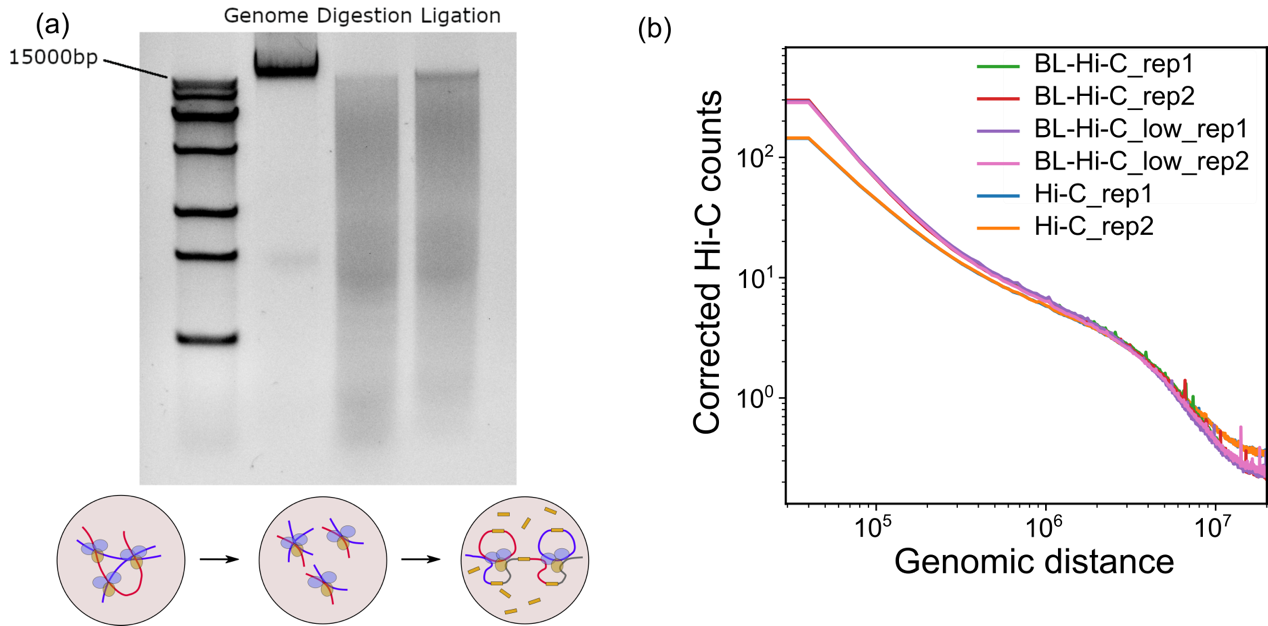


**Figure S1.** The quality control of BL-Hi-C. (a) Proof of principle experiment illustrating the BL-Hi-C. Agarose gel electrophoresis after nucleus digested with *Hea* III and ligated with bridge linker and genome DNA as a control. (b) Chromatin contact frequency (y-axis) was plotted as a function of linear genomic distance (x-axis) measured by BL-Hi-C and Hi-C in *B. rapa*.

**
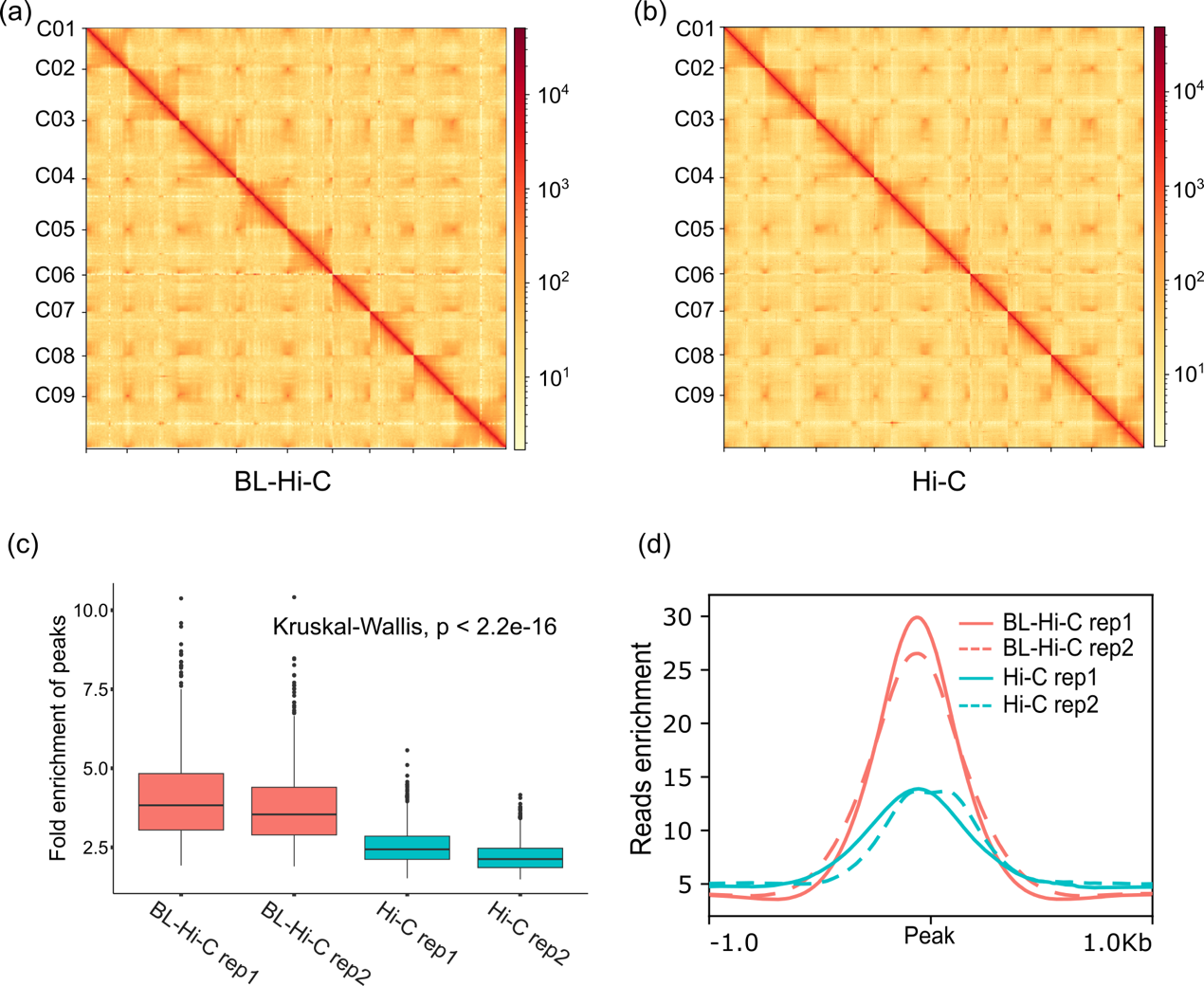
**

**Figure S2.** Comparsion of BL-Hi-C and Hi-C interactions in *B. oleracea*. (a-b) Genome-wide Hi-C maps at 500-kb resolution revealed by BL-Hi-C and Hi-C. (c) Boxplots showing the fold enrichment of peaks (n=1000). (d) Metaplot of signal enrichment around peaks.


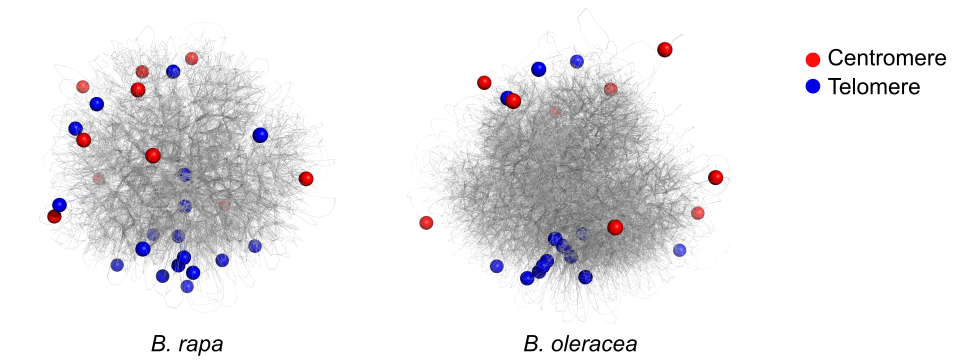


**Figure S3.** 3D organization of *B. rapa* and *B. oleracea* from BL-Hi-C

**
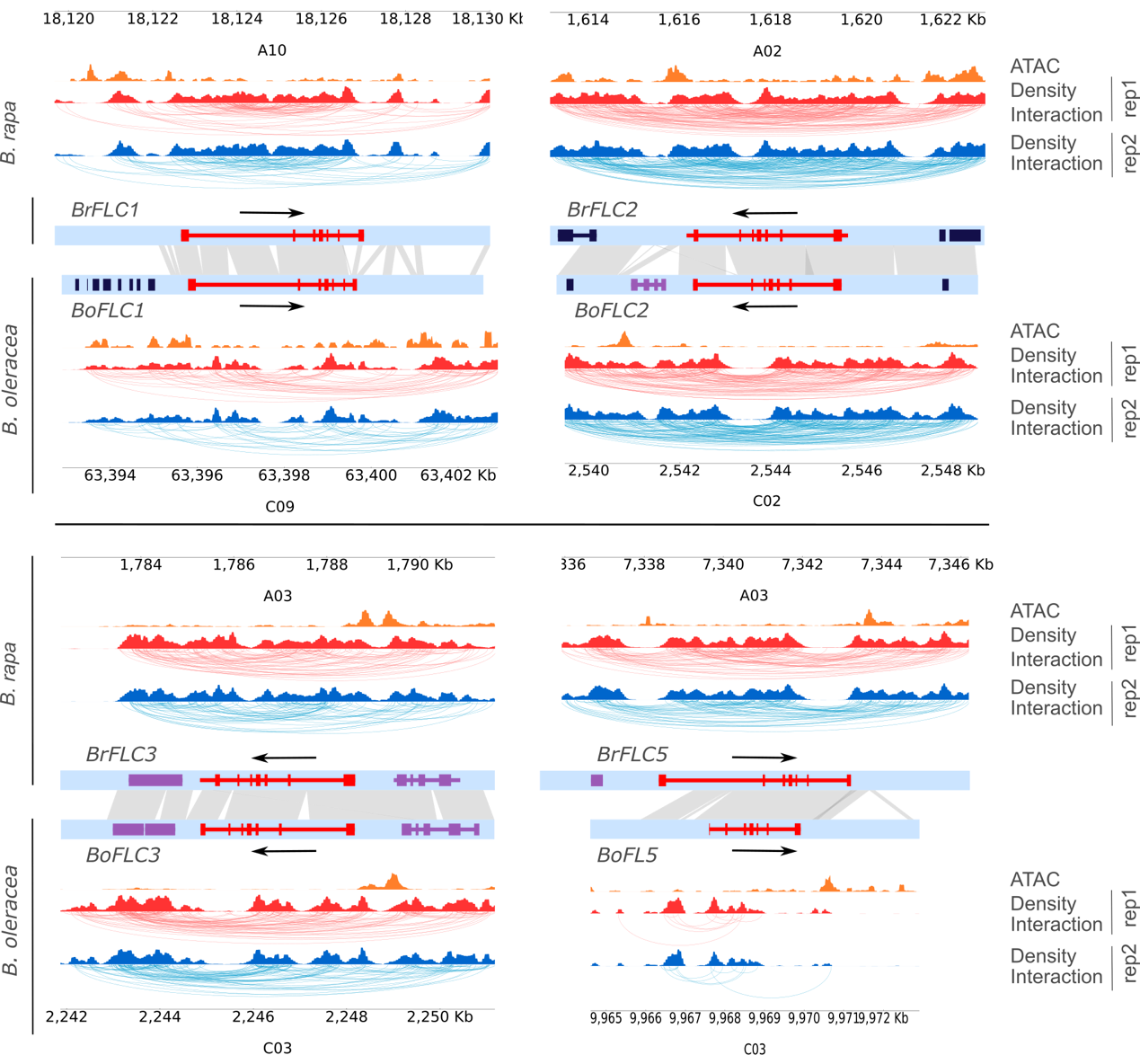
**

**Figure S4.** Comparison of *FLC* interactions in *B. rapa* and *B. oleracea* revealed by Hi-C. Syntenic analysis using 3 Kb regions upstream and downstream of genes.

**Table S2.** Cost comparison between BL Hi-C and Hi-C.

| Reagent | Supplier | Catalog | BL Hi-C  Cost($) | Traditional Hi-C Cost($) |
| --- | --- | --- | --- | --- |
| EGS | Thermo | 21565 | 3 | 0 |
| Hae III/Hind III | NEB | R0108L | 3 | 10 |
| Bridge linker | Sangon Biotech | / | 2 | 0 |
| dATP Solution/  Biotin-14-dCTP | NEB | N0440S | 1 | 170 |
| Klenow | NEB | M0212L | 3 | 10 |
| T4 ligase | NEB | M0202L | 6 | 40 |
| Lambda Exonuclease | NEB | M0262L | 2 | 0 |
| Exonuclease I | NEB | M0293L | 2 | 0 |
| Streptavidin Beads | Invitrogen | 65001 | 7 | 25 |
| AmpureXP beads | Beckman | A63880 | 23 | 5 |
| DNA Library Prep Kit |  | ND608-01 | 40 | 40 |
| Total | / | / | $92 | $300 |
